# Major upwelling and overturning in the mid-latitude *F* region ionosphere

**DOI:** 10.1038/s41467-018-05809-x

**Published:** 2018-08-20

**Authors:** David Hysell, Miguel Larsen, David Fritts, Brian Laughman, Michael Sulzer

**Affiliations:** 1 Department of Earth and Atmospheric Sciences, Cornell University, 2116 Snee Hall, Ithaca, NY 14853 USA; 2 Department of Physics and Astronomy, Clemson University, 140 Delta Epsilon Court, Clemson, SC 29634 USA; 3 GATS Inc., 3360 Mitchell Lane, Suite C, Boulder, CO 80301 USA; 4 Arecibo Radio Observatory HC 3, Box 53995, Arecibo, PR 00612-8346 USA

## Abstract

Profiles of the electron number density in the ionosphere are observed at the Arecibo Radio Observatory in Puerto Rico on a regular basis. Here, we report on recent observations showing anomalous irregularities in the density profiles at altitudes >~300 km. The irregularities occurred during a period of “mid-latitude spread *F*,” a space-weather phenomenon relatively common at middle latitudes in summer months characterized by instability and electron density irregularities in the bottomside of the ionospheric *F* layer. Remarkably, electron density irregularities extended well above the layer, through the ionization peak and into the topside which is regarded as being stable. Neither the neutral atmosphere nor the ionosphere is thought to be able to support turbulence locally at this altitude. A numerical simulation is used to illustrate how a combination of atmospheric and plasma dynamics driven at lower altitudes could explain the phenomenon.

## Abstract (web summary)

The ionosphere can give rise to irregularities that can interfere with radio communication, navigation and satellite systems. Here the authors present unique observations from the Arecibo Radio Observatory in Puerto Rico to reveal the existence of plasma irregularities occur in the mid-latitude *F*-region.

## Introduction

Since the 1960s, the Earth’s ionosphere has been studied with the incoherent scatter technique that uses radar to probe weak thermal fluctuations in the gas of free electrons in the upper atmosphere. The largest and most sensitive incoherent scatter radar is the 430-MHz system at the Arecibo Radio Observatory in Puerto Rico. At Arecibo, the plasma number density, ion composition, electron and ion temperature, and plasma drift velocity can be profiled by analyzing the backscatter from these thermal fluctuations. Early insights into the morphology of the ionosphere, its dependence on local time, season, and on the solar radiation flux came from pioneering incoherent scatter radar studies at Arecibo. The incoherent scatter technique was developed by a number of investigators^1–11^. A review of early ionospheric research at Arecibo was given by Mathews^12^.

It has long been appreciated that the ionosphere at both low and high magnetic latitudes can be unstable. Free energy bound in the configuration of ionospheric layers or in currents flowing within them can lead to the spontaneous growth of irregularities with spatial scale sizes ranging from tens of centimeters to thousands of kilometers. The irregularities can interfere with radio communication, navigation, and imaging systems, including satellite-based systems, and pose a hazard. Understanding, anticipating, and mitigating the effects are important aspects of space-weather research^13,14^.

It was not at first obvious that the mid-latitude ionosphere should be unstable since it is not subject to strong forcing from the magnetosphere as at auroral latitudes. Nor is the situation like that at equatorial latitudes where nearly horizontal magnetic field lines support the ionosphere against gravity, leaving it prone to certain kinds of plasma interchange instabilities. Two main classes of irregularities have been observed at middle latitudes, however. One is associated with dense layers of metallic ions that form in the *E* region of the ionosphere at altitudes near about 110 km. (The *E* region is the ionospheric layer in which Hall conductivity is significant, extending from about 95 to 150 km altitude.) These “sporadic *E*” or “E_*s*_” layers can become patchy or billowy, suggesting a causal role for Kelvin–Helmholtz instability (KHI) in the background neutral atmosphere^15,16^, although plasma instability has been suggested as a cause of the patchy layers as well^17^. Meter-scale density irregularities within the “E_*s*_” give rise to intense non-thermal Bragg scatter (called coherent scatter), which is visible to radar systems observing in the direction perpendicular to the Earth’s magnetic field^18–20^.

The other class of irregularities is associated with a phenomenon known as mid-latitude spread *F*. Here, “*F*” refers to the *F*-region ionosphere which is densest at altitudes >~250 km, and “spread” refers to spreading in visual representations of high-frequency radio soundings from the *F* region. This spreading was historically the earliest indication of plasma density irregularities in the mid-latitude *F* region (see ref. ^21^ for review).

Quiet-time spread *F* has been associated with thermospheric gravity waves and related undulations in the *F*-layer height, so-called medium-scale traveling ionospheric disturbances (MSTIDs)^22–26^. It has also been linked by numerous investigators to a particular plasma instability mechanism thought to operate at middle latitudes attributed to Perkins^27^. Mid-latitude spread *F* has furthermore been associated with patchy E_*s*_ layers, observationally and theoretically, although different perspectives exist regarding the causal relationship^28–32^. Meter-scale irregularities in the *F* region are also known to cause coherent scatter^33,34^. Coherent scatter is a useful tool for identifying plasma instability and ionospheric irregularities but does not measure plasma state variables like incoherent scatter does. The relationship between coherent scatter and the condition of the ionosphere that produces it is indirect^35^.

We show here observations of mid-latitude *F*-region plasma density irregularities occurring in tandem with E_*s*_-layer irregularities over Arecibo during a geomagnetically quiet period. The morphology of the irregularities together with quantitative information about the underlying plasma state and dynamics are captured precisely in the Arecibo data. Previous measurements of mid-latitude spread *F* at Arecibo and elsewhere have suggested wavelike variations in the height of the *F*-layer peak. The observations presented here are unique in that they show much deeper instability with significant upwelling and overturning in plasma density extending well into the topside. We also present coherent scatter echoes from a common radar scattering volume in the *E* region observed nearby from St. Croix. The measurements reveal unexpected aspects of the irregularities and provide new clues about the instability mechanisms at work.

We present the results of a numerical simulation of the mid-latitude ionosphere which explains the most important features in the Arecibo observations including the patchy E_*s*_ layers, MSITDs, bottomside spread *F* irregularities, and irregularities in the topside. The most important ingredient in the simulations are intense gravity waves propagating upward into the thermosphere. The gravity wave wind fields both form the E_*s*_ layers and cause them to structure, induce MSTIDs through dynamo action, and drive currents in the *F* region which lead to the formation of bottomside irregularities. Most importantly here, the gravity waves induce electric fields in the *E* region which map along magnetic fields into the topside where they produce irregularities in the topside through convection and advection.

## Results

The results of this paper are divided into radar observations from Arecibo and numerical simulations meant to help interpret the observations. These are addressed separately.

### Observations

**Fig. 1:**
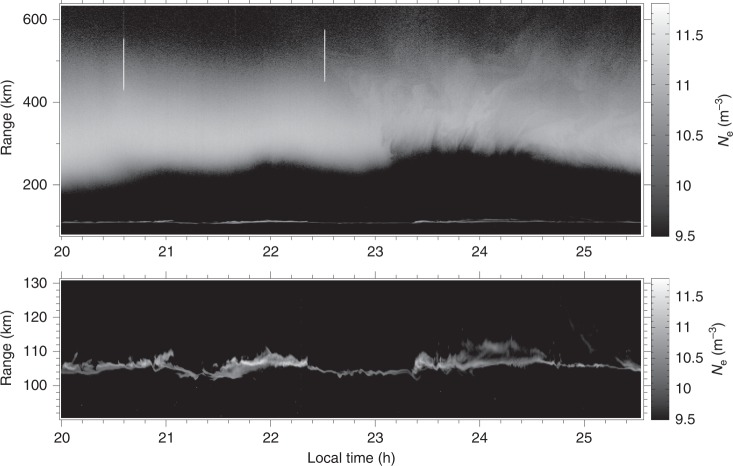
Electron number density on the evening of 30 July 2016, represented as a function of altitude and local time in grayscale format. The figure shows both the *E* and *F* regions on the same scale and the *E* region in an expanded scale

Ionospheric observations were made with the Arecibo Observatory on 30 July 2016, using the incoherent scatter technique and a mode which was described in detail by Hysell et al.^36^. The results are shown in Fig. 1. The figure shows electron number density versus altitude and local time directly over the radar. The experimental cadence is one profile per minute, and the range resolution is 300 m. In processing these data, a single noise estimate for the entire period shown was calculated and subtracted from the receiver power at all times. This procedure introduces a small bias since the noise estimate does not capture the noise variation with sidereal time. However, it greatly reduces the temporal variance of the resulting plasma number density estimate, revealing minute variations that would otherwise be difficult to distinguish.

The bottomside of the *F* layer is modulated with a period of roughly 60–90 min. This is a common ionospheric feature, termed MSTID, which is usually attributed to gravity waves in the thermosphere. The modulation crests have a sawtooth appearance. The third crest of the MSTID is moreover populated with narrow plasma density depletions spanning altitudes between ~250 and 350 km and extending up through the peak of the *F* layer. These irregularities are characteristic of mid-latitude spread *F* conditions and indicative of plasma instability. Note that the depletions are tilted from vertical.

More plasma irregularities were observed in the *E* region where an E_*s*_ layer was seen to erupt into dense patches. In this case, the patches were approximately coincident in time with the crests in the MSTID. Only the third set of patches was coincident in time with mid-latitude spread *F*, however. Note that the *E-* and *F*-region ionization directly above Arecibo, where the magnetic dip angle is approximately 44°, does not reside on common magnetic field lines, and no claim can be made regarding the electrical coupling of the *F* region and the E_*s*_ layers. Experience at Arecibo has shown that patchy E_*s*_ layers, MSTIDs, and spread *F* can occur together or independently in time.

**Fig. 2:**
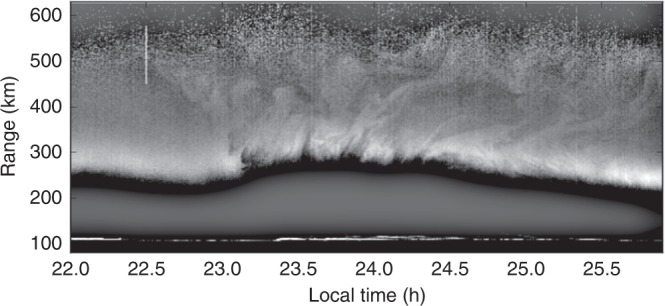
Enhanced version of the topside ionospheric irregularities from the latter portion of the event. A low-pass filter (Gaussian with a standard deviation of 100 pixels) was applied to the original image, and the difference between the filtered and unfiltered images was then plotted. Despite having units of per cubic meter, the quantity being plotted is not strictly electron density, and the grayscale is arbitrary

What is most remarkable about the observations are the faint plasma density irregularities in the topside ionosphere, >~350 km, visible from ~2230 LT through the end of the observation. The irregularities are more evident in Fig. 2 where they have been enhanced. The irregularities exist throughout the topside and appear to modulate the topside boundary. They are both wavelike and, at least superficially, turbulent. They both precede the striated depletions in the bottomside *F* region in time and linger behind them for at least an hour.

These irregularities are remarkable in that the topside ionosphere is usually regarded as laminar and stable. The neutral gas as these altitudes is highly viscous and unable to support inertial-range turbulence. Ion inertia is also usually regarded as negligible in this regime of space, implying that the plasma also cannot support inertial-range turbulence. It is, therefore, somewhat surprising for the topside to exhibit telltale signs of turbulence like this. As noted above, coherent scatter has been observed from the topside mid-latitude ionosphere in the past during spread-*F* events, signifying plasma instability. What is new here is the observation of superficially turbulent density irregularity and flow in the topside revealed by incoherent scatter.

The incoherent scatter technique affords measurements of other state parameters in the ionosphere such as ion composition, temperature, and line-of-sight drifts. There are two feed systems that can probe the ionosphere along two distinct lines of sight. For these experiments, one (the line feed) was pointed at zenith, and the other (the Gregorian feed) at 15° zenith angle and at azimuth angles swept between 270° and 360°. From dual-beam data, estimates of the vector drift velocity of the plasma can be made with the assumption that the flow is spatially homogeneous over the volume probed by the two beams. Vector wind profiles can furthermore be estimated in the *E* region using statistical inverse methods, invoking the conservation properties of the plasma and assuming that the components of the electric field transverse to the magnetic field in the *E* and *F* region are the same. The methods involved were described in detail by Hysell et al.^36^.

**Fig. 3:**
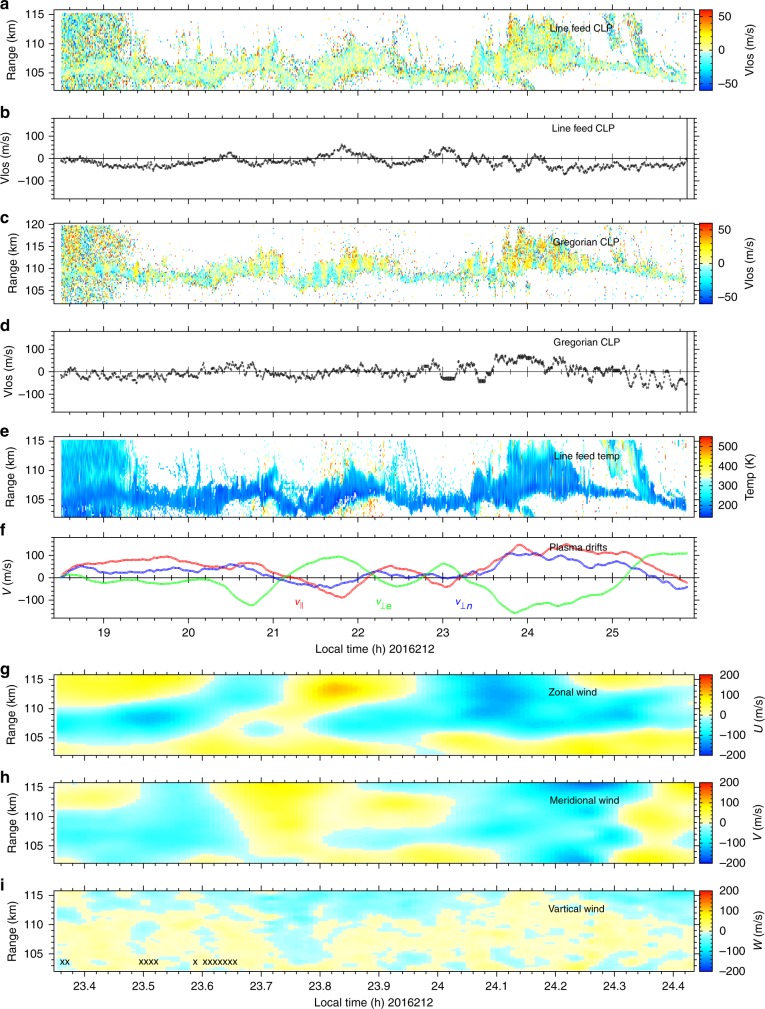
State parameters derived from incoherent scatter measurements using dual radar beams. **a** Line-of-sight *E*-region plasma drift profiles measured at zenith. **b** Average line-of-sight *F*-region plasma drifts measured at zenith. **c** Line-of-sight *E*-region plasma drift profiles measured off zenith. **d** Average line-of-sight *F*-region plasma drifts measured off zenith. **e** *E*-region temperature profiles. **f** Average *F*-region vector plasma drifts. Here the red, green, and blue lines indicate drifts parallel to the geomagnetic field, perpendicular and eastward, and perpendicular and upward/northward, respectively. **g** *E*-region zonal wind profiles. **h** *E*-region meridional wind profiles. **i** *E*-region vertical wind profiles. The bottom three panels reflect conditions within just the third patchy E_*s*_ layer

Figure 3 shows the results of the aforementioned analysis. We highlight the results of panel (f) which shows estimates of the vector plasma drifts in the *F* region. The red, green, and blue curves denote drifts parallel to the magnetic field **B**, perpendicular to **B** and eastward, and perpendicular to **B** and northward, respectively. That the difference between the red and blue curves is generally small signifies the fact that the upward drifts were most often modest, echoing the information in panel (b). The average of the red and blue curves gives the northward drift which was large and oscillatory. The green curve gives the eastward drift which was also large and oscillatory. During much of the mid-latitude spread *F* event, the *F* region was drifting rapidly (>150 ms^−1^) to the northwest.

Panels (g) and (h) show estimates of the horizontal winds in the *E* region. As is typical, the winds are large (~100 ms^−1^), and the horizontal winds in particular are characterized by strong shears. Similar observations led^37^ to propose neutral KHI as the mechanism underlying E_*s*_ layer patchiness.

Additional contextual information comes from a small 29.8-MHz radar system located on St. Croix, USVI. This radar detects coherent backscatter from meter-scale plasma density irregularities embedded in the E_*s*_ layers. The irregularities in question are strongly elongated in the direction of the magnetic field **B**. The radar is located so as to be able to detect strong Bragg or coherent scatter from *E*-region irregularities directly over Arecibo. The radar nominally uses 28-bit Barker coded pulses with a baud length of 7 μs and a pulse repetition frequency of 400 Hz. The incoherent integration time is nominally 3 s.

**Fig. 4:**
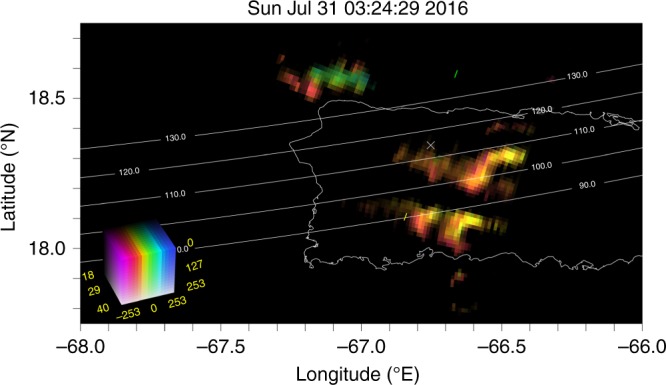
Representative images of radar echoes due to coherent scatter from plasma density irregularities in E_*s*_ layers near 110-km altitude at 2324.5 LT. The brightness of the image pixels specifies the echo signal-to-noise ratio on a decibel scale. The hues reflect Doppler velocity, with red (blue) hues denoting drifts away from (toward) the radar located on St. Croix, USVI

Figure 4 shows a plan view of the backscatter seen at St. Croix during the mid-latitude spread *F* event. Images like these are constructed using aperture synthesis methods of the kind used by radio astronomers to image distant radio sources in the cosmos. Images can be computed at a cadence of once every few seconds. The imaging technique utilized here, which has been described in detail by Hysell and Chau^38^, gives information about the Doppler shift of the echoes as well as the intensity of the backscatter. The velocities indicated by the color scale are the phase velocities of the meter-scale waves in the plasma which is controlled by a number of factors, the proper motion of the E_*s*_ layer patches being only one of them.

Studies have shown that the strongest coherent backscatter generally arises from the densest regions of the patchy E_*s*_ layer as determined by incoherent scatter. The coherent scatter therefore conveys information about the horizontal configuration of the patches, their proper motion, their propagation, evolution, and lifetimes. This image suggests that patchy E_*s*_ layers extended well away spatially from the immediate vicinity of Arecibo.

This representative image shows that the E_*s*_-layer patches are organized horizontally along fronts. In this case, there are two fronts over Puerto Rico and a third to the northwest. The two larger fronts are themselves structured, showing evidence of secondary waves along their length. When animated, successive images from this event show that the fronts propagate in space in different directions but most often to the southwest. This is typical of E_*s*_-layer observations made using this instrument. Fronts are typically separated by a few tens of kilometers, propagate at about 100 ms^−1^, and exhibit periods of about 5 min. It has been argued by Larsen^37^ and Hysell et al.^39^ that the fronts represent individual Kelvin–Helmholtz billows which deform E_*s*_ layers that were originally produced and then broken up by the neutral shear flow. In this scenario, *E*-region coherent scatter is indicative of strong winds and wind shears in the lower thermosphere.

### Numerical simulation

We can attempt to reproduce the ionospheric irregularities observed over Arecibo, the *F*-region and topside irregularities in particular, through numerical simulation. The simulation couples together two models—one of neutral thermospheric dynamics, and another of the ionospheric plasma response. Neutral winds predicted by the former are used to drive ionospheric evolution in the latter.

The numerical simulation is described in more detail in the methods section of this paper. The problem is complex, and a number of compromises have been made in the modeling in order to keep the computations tractable. For example, the neutral dynamics model is cast in two dimensions only. In previous model studies, primary KHIs with two-dimensional (2D) symmetry arise and achieve large amplitudes before 2D and three-dimensional (3D) secondary instabilities which break the symmetry and go on to affect billow dissipation^40,41^. By modeling the neutral dynamics in 2D, we will be testing whether the multifaceted ionospheric phenomena we observe can arise from relatively simple 2D neutral wind forcing. The results are extended into three dimensions by assuming invariance in the unmodeled horizontal dimension. When the neutral wind field is introduced into the ionospheric model, the unmodeled dimension is offset by 5° from the magnetic meridian. This figure is arbitrary and can be varied without significantly affecting the simulation outcome.

Another limitation of the simulation is that it neglects reverse coupling from the ionosphere back to the neutral atmosphere. The ionospheric plasma is only a minor constituent of the upper atmosphere and represents a small part of the momentum budget. While ion drag alters the neutral atmospheric circulation at low and middle latitudes as well as the temperature and composition, (e.g., ref. ^42^ and references therein), the timescale for altering the neutral circulation is proportional to the ratio of neutral and plasma mass density and is of the order of hours in the mid-latitude *F* region^43^. This is long compared to the e-folding time of the plasma instabilities of interest here. We nonetheless cannot fully discount a role for reversed coupling which could include thermal and compositional effects. A fully consistent treatment of the coupled neutral/plasma dynamics, thermodynamics, and chemistry is a priority for future research, but is beyond the scope of the present analysis.

A third limitation of the simulation is that it does not make provisions for ionized metallic species. Sporadic *E* layers are known to be composed primarily of metallic ions, the small rates of diffusion and recombination of metallic species being important contributing factors to the intensity of the layers which can rival the *F* peak in density. This simulation cannot reproduce very dense E_*s*_ layers. We are therefore essentially testing in simulation whether dense E_*s*_ layers are a necessary component of the ionospheric plasma instabilities underlying mid-latitude spread *F*. The results presented below suggest that they are not.

**Fig. 5:**
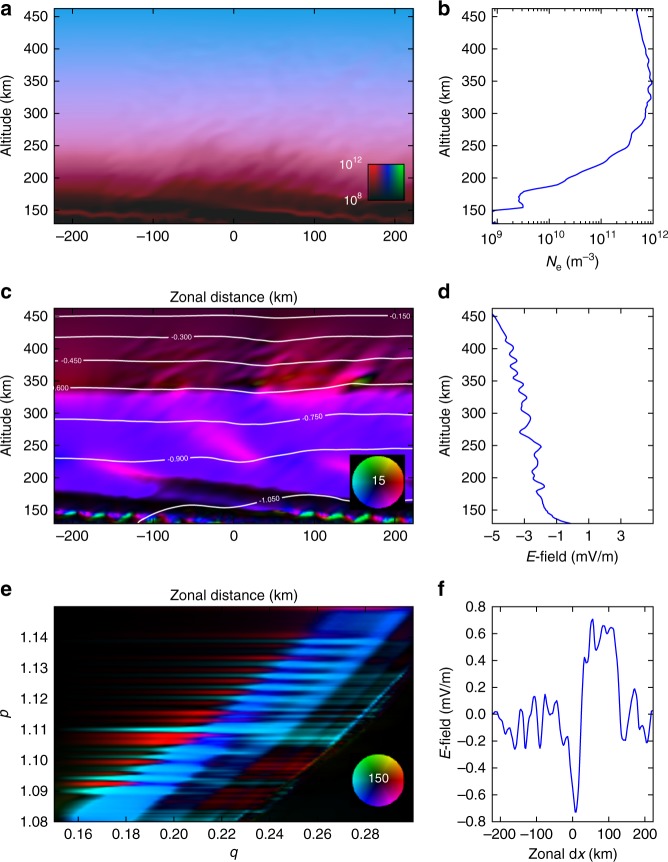
Results from the ionosphere simulation after 25 min of ionospheric evolution. **a** Plasma number density, with red, blue, and green hues representing molecular ions, atomic ions, and protons, respectively, in the plane perpendicular to **B** in a 2D cut through the center of the model volume. **b** Electron density profile through the center of the top left panel. **c** Current density in μA m^−2^ according to the color wheel, also in a perpendicular-to-**B** cut through the center of the model volume. White contours are equipotential curves which are streamlines of the flow. **d** Electric field profile through the center of the middle left panel. **e** Meridional current density in μA m^−2^ according to the color wheel. **f** Zonal electric field in the equatorial plane in a cut through the center of the model volume. The color wheels indicate the magnitude and direction of the current density relative to the maximum value of either 15 or 150 nA m^−2^ full scale

Figure 5 shows results of the numerical simulation 25 min into a run initialized at 2330 local time. (The start time of the ionospheric model is 142 min after gravity wave (GW) initialization.) Key features of the ionospheric plasma configuration include the sawtooth modulation of the *F*-region bottomside which is like that of an MSTID. This is a consequence of the thermospheric gravity wave packet which induces periodic plasma motion through dynamo action. In the middle left panel of Fig. 5, nearly horizontal streamlines indicative of westward flows at high altitudes become more strongly modulated in the bottomside, even forming closed loops (vortices) at times. Despite the strong modulation of the plasma flow, however, the bottomside deformation remains limited. This is because the gravity wave phase speed does not match the plasma flow speed, and over time, any given plasma volume is likely to ascend and descend alternately as the gravity wave propagates through. This phenomenon, known as spatial resonance, prevents MSTID breaking. The direction of the net plasma flow also changes from westward to eastward over time as the background winds shift. Similar behavior was evident in panel (f) of Fig. 3 which shows several reversals in the zonal plasma flow.

Another feature reproduced by the simulation is the appearance of patchy E_*s*_ layers in the lower thermosphere. The layer forms at the node of the neutral wind shear over time, i.e., there was no thin layer in the model initial conditions. Neutral dynamic instability and the attendant plasma structuring are responsible for the patchiness. The layer in the model is composed of molecular ions, and the amplitude is limited by a combination of diffusion and recombination. In nature, the layer could be composed of metallic ions, permitting it to become much denser.

Other signature features of the simulation are tilted striations in the bottomside concentrated near the MSTID crest. The striations resemble the narrow density depletions seen in the bottomside in the mid-latitude spread *F* event if we roughly equate space and time and consider relatively steady flow during the period the striations passed over the radar. They arise in the simulation where the transverse (to **B**) current density has a significant vertical component and the vertical plasma density gradient is steep. The striations are very similar to irregularities, which have been predicted analytically and produced in numerical simulations of the bottomside equatorial *F*-region ionosphere^44–46^. The mechanism at work in the equatorial case is termed collisional shear instability since plasma shear flow is necessarily associated with it. In the equatorial analyses and simulations, density striations immersed in a background wind field become electrically polarized in such a way as to satisfy the quasineutrality condition, and the resulting plasma flow increases the amplitude of the striations. The growth rate of this instability is maximum for irregularities that are oriented neither horizontally nor perpendicular to the horizontal but at an intermediate angle. The mechanism functions in the mid-latitude simulation as well and so could be a key component of mid-latitude spread *F*.

The lower left panel of Fig. 5 shows currents flowing in the plane of the magnetic meridian. Constant-*p* surfaces follow magnetic field lines, and *q* is related to magnetic latitude^47^. Currents flowing to the right and left are field-aligned currents. Transverse (to the magnetic field) currents can be driven both by irregular winds and by winds blowing in regions where the plasma conductivity is irregular. Field-aligned currents then flow between different regions to maintain quasineutrality in the plasma. The figure shows how different ionospheric layers are coupled by the field-aligned currents which connect them. The field-aligned currents shown here flow from the *E* region through the *F* peak and into the topside ionosphere. The conductivity is distributed evenly enough in altitude to allow current sources in all the altitude strata to influence the plasma dynamics globally. This allows winds in the lower thermosphere to drive plasma dynamics in the topside.

Indeed, Fig. 5 shows evidence of plasma density irregularities in the topside ionosphere. These are seen faintly in panels (a) and (b). The evidence is more clear in panel (c) which shows irregularities in current density in topside strata that happen to be above the strongest concentrations of plasma and neutral instabilities. The sources of the irregularities are (1) winds blowing across the bottomside plasma irregularities where the conductivity is irregular and (2) turbulent winds in the lower thermosphere where KHI is starting. Both factors disrupt the transverse plasma flow globally. Topside density irregularities may then be generated where the irregular flow stirs the topside background plasma density gradient. This could be how topside plasma irregularities come into being at middle latitudes where magnetic field lines subtend a wide range of altitudes.

**Fig. 6:**
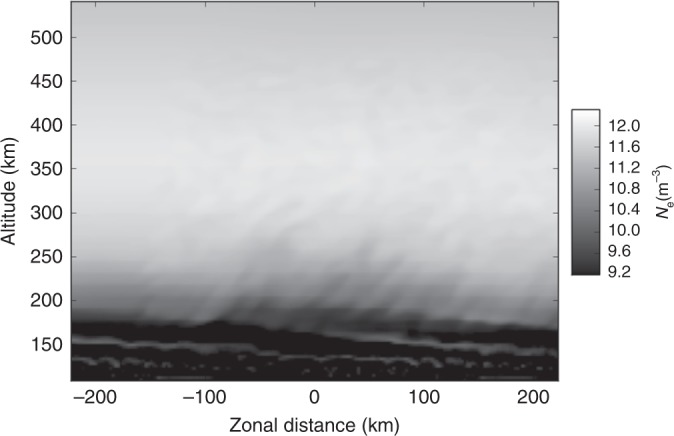
Detailed view of a vertical cut through the simulated electron density after 25 min of simulation time in grayscale format

Figure 6 shows the electron density from the simulation at the same time as the previous figure. In this figure, however, the vertical axis represents altitude taken along a purely vertical cut through the simulation. The electron density is also shown in grayscale format, promoting a comparison with the Arecibo ISR data. To convert from the spatial axis of Fig. 6, we can take the zonal flow speed in the simulation to be about 100 ms^−1^, meaning that it would take approximately 75 min for the simulated structure to pass over a fixed site assuming steady, frozen flow. In the figure can be found the most important features of the ISR dataset including, crucially, electron density fluctuations extending well into the topside (>~350 km altitude in the case of the simulation).

## Discussion

The Arecibo dataset presented here points to a number of ionospheric phenomena, indicators of several distinct but related driving mechanisms. The first is gravity waves propagating in the thermosphere. In simulation, a gravity wave wind field induces ionospheric currents, and because the conductivity is inhomogeneous, the ionosphere becomes polarized and deforms, resulting in periodic undulations or MSTIDs. The amplitude of the MSTIDs is limited by the fact that the wind field varies along magnetic field lines which act like equipotential lines, “shorting out” the polarization to some degree. The amplitude is also limited by the fact that the gravity waves are not frozen in the frame of reference of the ionospheric plasma. Parcels of ionospheric plasma ascend and then descend under the action of the wave, and the MSTID does not break.

The currents induced by the gravity wave wind field are moreover able in simulation to drive plasma instabilities with scale sizes much smaller than the MSTIDs. That the zonal winds and the zonal plasma drifts are different implies the existence of Pedersen current with a vertical component. This current can excite interchange-type plasma instabilities, i.e., instabilities that occur where current flows transverse to the magnetic field in regions where the plasma is inhomogeneous^48^. Irregularities produced by this mechanism in simulation resemble the irregularities seen by Arecibo associated with mid-latitude spread-*F* conditions. The irregularities are confined mainly to the bottomside *F*-region ionosphere and tilted from vertical. The mechanism at work appears to be similar to the collisional shear instability associated with the bottomside equatorial *F*-region ionosphere. Small-scale irregularities embedded within them could produce scintillation in transionospheric radio signals and result in degradation of radio communication, navigation, and imaging systems.

In the numerical simulation and in nature, additional structuring in the topside ionosphere emerged where neither neutral nor plasma instability is expected to operate locally. The structuring seen over Arecibo appears to be turbulent, at least superficially, a characteristic which is unexpected in flows in both the neutral and charged gasses at upper thermospheric altitudes.

The plasma circulation all along a given magnetic field line is controlled by neutral wind dynamos at altitudes where the Pedersen conductivity is significant and where either the plasma conductivity or the wind itself is highly irregular. The conductivity is irregular in the MSTID crests where interchange-type plasma instability is active, and the winds can be highly irregular in the lower thermosphere where roll-like deformations in E_*s*_ layers suggest the presence of KHI. We attribute the anomalous topside ionospheric irregularities to a combination of these two mid-latitude phenomena which can induce subtle irregularities far from where they are immediately located.

## Methods

A numerical simulation of the Arecibo dataset described in this paper was constructed in order to investigate some of the factors possibly responsible for the bottomside and topside *F*-region plasma density irregularities observed. The simulation involves a model of neutral thermospheric dynamics driving a model of ionospheric plasma dynamics.

### Neutral atmosphere dynamics

Neutral atmosphere dynamics are described by a 2D finite-volume (FV) code that solves the anelastic Navier–Stokes equations in a horizontally periodic Cartesian domain extending well into the thermosphere. Previous studies have demonstrated its ability to describe GW propagation and nonlinear dynamics in 2D and 3D extending into the thermosphere and ionosphere in a variety of applications^49–53^.

The FV code formulation retains density fluctuations only in the buoyancy term and employs a thermodynamic definition of the potential temperature fluctuation that is modified to achieve an equation set that conserves mass, momentum, total energy, and potential vorticity, apart from dissipative effects^54–56^. Assuming a relatively high-frequency GW such that rotation is negligible, the governing equations may be written as:

1 $$\frac{{\partial \bar \rho u_j}}{{\partial x_j}} = 0,$$

2 $$\frac{{\partial \bar \rho u_i}}{{\partial t}} + \frac{{\partial \bar \rho u_iu_j}}{{\partial x_j}} = - \frac{{\partial p\prime }}{{\partial x_i}} + \left( {\frac{{\bar \rho {\mathrm{\Theta }}\prime g}}{{\overline {\mathrm{\Theta }} }} - \frac{{p\prime }}{H}} \right)\delta _{i3} + \frac{\partial }{{\partial x_j}}\left[ {\mu \left( {\frac{{\partial u_i}}{{\partial x_j}} + \frac{{\partial u_j}}{{\partial x_i}}} \right)} \right],$$

3 $$\frac{{\partial \bar \rho {\mathrm{\Theta }}}}{{\partial t}} + \frac{{\partial \bar \rho {\mathrm{\Theta }}u_j}}{{\partial x_j}} = \frac{{{\bar{\mathrm \Theta }}}}{{c_p\bar T}}\left[ {\mu \left( {\frac{{\partial u_i}}{{\partial x_j}} + \frac{{\partial u_j}}{{\partial x_i}}} \right)\frac{{\partial u_i}}{{\partial x_j}} - \frac{\partial }{{\partial x_j}}\left( {\kappa \frac{{\partial \bar T}}{{\partial x_j}}} \right)} \right].$$

Here, overbars denote mean fields, primes denote deviations from these fields (e.g., $p = \bar p + p\prime$), and the solution variables are the velocity, *u*_*i*_ or (*u*, *v*, *w*), the pressure fluctuation, *p*′, and the potential temperature, *θ*. Equations (1)–(3) are discretized using a second-order, FV scheme yielding exact numerical conservation of mass, momentum, and kinetic and thermal energy (apart from explicit dissipation) and thus faithfully represents the underlying conservation laws^49^. In the above, *μ* = *νρ* is the dynamic viscosity, *ν* is the kinematic viscosity, *ρ* is the mass density, and *κ* is the thermal diffusivity. We assume *ν/κ* = 1 so that the thermal and velocity fields require the same resolution.

Equations (1)–(3) yield a dispersion relation for linear, inviscid, steady GW motions in a non-rotating, isothermal atmosphere with uniform *U* and *N* that is identical to that for the GW branch of the compressible acoustic-GW dispersion relation^56^ given by

4 $$\omega _i^2 =	 \frac{{N^2}}{{1 + (m/k)^2 + [1/(2kH)]^2}}$$

5 $$\qquad\quad =	 \frac{{N^2}}{{1 + (\lambda _x{\mathrm{/}}\lambda _z)^2 + [\lambda _x{\mathrm{/}}(4\pi H)]^2}}.$$

A radiation condition is imposed at the upper boundary that is an anelastic extension of the Klemp and Durran method^57^, modified such that the required polarization relations are assessed from the computed solution near the upper boundary rather than being specified a priori. This modification allows the radiation condition to indirectly account for viscous and nonlinear effects near the upper boundary, which can lead to polarization relations that differ significantly from the predictions of linear inviscid theory. The FV code also allows a “sponge” layer at the upper boundary to ensure no GW reflection in cases where large incident amplitudes may violate the assumed linear radiation condition. Horizontal boundary conditions are periodic.

**Fig. 7:**
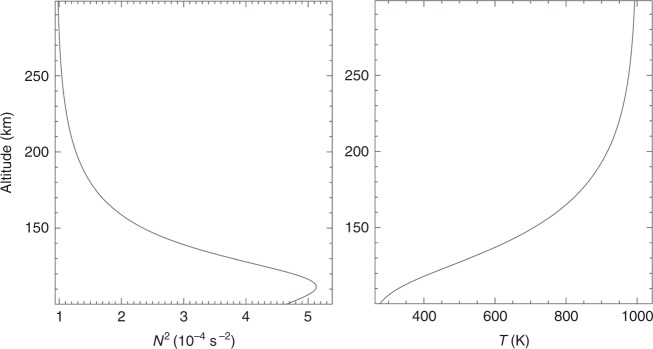
Profiles of *N*^2^ and *T* employed for the GW simulation. *N*^2^ and *T* at lower altitudes asymptote to 4 × 10^−4^ s^−2^ and 240 K, respectively

We assume GW propagation in an (*x*, *z*) plane without loss of generality and perturbations having the form *ϕ*′ = $\phi _ \circ ^\prime {\mathrm{exp}}[i(kx + mz - \omega t)]$ with a specified horizontal wavelength *λ*_*x*_ = 2*π*/*k* = 300 km, initial vertical wavelength *λ*_*z*_ = 2*π*/*m* = 40 km, and frequency *ω* = 0 at the source level in the reference frame moving with the GW horizontal phase velocity *c*. These parameters are roughly consistent with the characteristics of typical MSTIDs observed in the upper atmosphere. We also assume a background environment initially at rest at all altitudes having constant buoyancy frequency, *N* = 0.02 s^−1^, mean temperature, *T*(*z*) = 240 K, and scale height, *H* = 7 km, at lower altitudes, with *T* and *N*^2^ varying in the thermosphere as shown in Fig. 7 and *N*^2^ = (*g*/*T*)(d*T*/d*z* + *g*/*c*_*p*_), where *g*, *T*, and *c*_*p*_ are gravitational acceleration, temperature, and specific heat at constant volume, respectively.

An initial GW packet having a Gaussian amplitude variation in altitude with a full-width/half-maximum of 50 km was specified such that amplitude growth as *ϕ*′ ~ *e*^*z*/2*H*^ yielded a large GW amplitude, KHI, and irreversible induced mean flows beginning at ~105 km. The simulation employed a spatial resolution of 500 m over a domain extending 450 km in the vertical and 300 km in the horizontal, in order to resolve these dynamics.

Mean and GW parameters specified at lower altitudes imply an initial GW phase speed *c* = 127 ms^−1^. Variations in *T* and *N*^2^ in the thermosphere and variations in *U* and *c* accompanying GW momentum transport, mean-flow accelerations, and GW self-acceleration^50^, imply significant variations of the GW *λ*_*z*_, *c*, and vertical phase structure with time and altitude. The induced mean flow is given by d*U*/d*t* = −(1/*ρ*)d(*ρ*〈*u*′*w*′〉)/d*z*, where 〈*u*′*w*′〉 is the GW momentum flux and 〈〉 denotes an average over GW phase. The GW intrinsic frequency, *ω*_*i*_ = *k*(*c* − *U*), is related to the GW and mean parameters through Eq. (4), with the mean wind, *U* = −*c*, specified such that the GW is steady in the ground-based reference frame in the absence of mean flow accelerations. Note that *U* changes are only permanent accompanying GW dissipation and are reversible if d*U*/d*t* is due entirely to GW localization and transience.

**Fig. 8:**
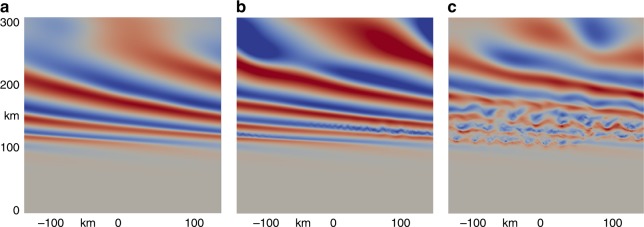
Cross-sections of GW and KHI *u*′. At **a** 138, **b** 155, and **c** 172 min after initiation of the GW packet at lower altitudes. Maximum *u*′ is approximately 150 ms^−1^. The dominant KHI horizontal scales at the final time are ∼30–40 km

The GW evolution is shown with contours of *u*′ at three times from ~138 to 172 min in Fig. 8. GW propagation is upward and to the left in these panels. At the earliest time, the GW has not yet achieved it maximum amplitude (nor initial KHI) between ~100 and 150 km, but it has experienced vertical phase compression (reductions in *λ*_*z*_) below and above 100 km accompanying transient *U* accelerations that impose a smaller *c* − *U* on the trailing GW packet. Continued GW amplitude increases by 155 min (middle panel in Fig. 8) reveal further vertical phase compression and initial, small-scale KHI accompanying intensifying GW wind shears. The KHI spatial scales and amplitudes increase dramatically over the next ~17 min as the GW achieves it maximum amplitude at these altitudes. These are the times at which these neutral dynamics yielded strong responses in the ionospheric model code, so the further evolution of this flow is not of interest.

These results are of 2D neutral dynamics, where in reality, KHI would yield 3D secondary instability structures at sufficiently high Reynolds numbers. However, the same response would be virtually guaranteed using a 3D model resolving 3D KHI dynamics. Additionally, high Reynolds numbers for this event likely would enable 3D secondary instabilities, but not by a wide margin. But 3D results by themselves could have led to the conclusion that the 3D plasma responses were due to 3D neutral dynamics, which is clearly not the case here. This suggests that the plasma dynamics themselves evolve 3D structures with 2D neutral forcing.

The simulated wind field is extended to three dimensions with the assumption that the winds are invariant in the unmodeled horizontal direction. The resulting wind frame of reference is then rotated by 5° and then coupled into the ionosphere model. (The rotation is not essential but was introduced to allow for the possibility of winds in the meridional direction.)

### Plasma dynamics

The neutral winds are used to drive a separate model of mid-latitude ionospheric evolution. The model considers the dynamics of four ion species (O^+^, NO^+^, O2^+^, and H^+^) plus electrons. It evolves the number density of these species by enforcing the conservation of number density and momentum. Limited chemistry including charge exchange and dissociative recombination is considered. The momentum equation can be solved explicitly in the collisional regime relevant here, yielding the following expression for the velocities of the charged species:

6 $${\bf{v}}_s - {\bf{u}} = \mu _s \cdot ({\bf{E}} + {\bf{u}} \times {\bf{B}}) - {\bf{D}}_s \cdot \nabla {\kern 1pt} {\mathrm{ln}}{\kern 1pt} n_s + {\bf{T}}_s \cdot {\bf{g}},$$

where *μ*_*s*_, **D**_*s*_, and **T**_*s*_ are the tensor mobility, diffusivity, and gravitational coefficients for species *s*, which can be evaluated using state-parameter estimates taken from empirical models (see below) following prescriptions found in ref. ^58^. Combining the velocities gives a specification for the current density **J** ≡ $\mathop {\sum}\nolimits_s \left( {q_sn_s{\bf{v}}_s} \right)$ of the form

7 $${\bf{J}} = \sigma \cdot ({\bf{E}} + {\bf{u}} \times {\bf{B}}) - \mathop {\sum}\limits_s {\kern 1pt} q_s{\bf{D}}_s \cdot \nabla n_s + {\mathrm{\Xi }} \cdot {\bf{g}},$$

where *σ* is the conductivity tensor and Ξ is a tensor specifying the current density driven by gravity. Expressions for the tensors in the case where Coulomb collisions are neglected were given by Shume et al.^59^. The effects of Coulomb collisions are included in the parallel transport in the current, more generalized form of the model in which collision terms coupling the ion species in Eq. (6) also appear.

We divide the electric field into a background and a perturbed component, viz., **E** = **E**_ο_ − ∇*ϕ*, and solve the resulting elliptic partial differential equation for the electrostatic potential *ϕ*. The model solves for the potential in three spatial dimensions by imposing the quasineutrality condition, i.e., the condition that the number density of ions and electrons be equal throughout the simulation. This is equivalently the condition that the current density be everywhere solenoidal. Currents driven by electric fields, winds, pressure gradients, and gravity are included in the model as shown above. The potential equation implied by the quasineutrality condition,

8 $$\nabla \cdot (\sigma \nabla \phi ) = \nabla \cdot \left[ {\sigma \cdot \left( {{\bf{E}}_ \circ + {\bf{u}} \times {\bf{B}}} \right) - \mathop {\sum}\limits_s {\kern 1pt} q_s{\bf{D}}_s \cdot \nabla n_s + {\mathrm{\Xi }} \cdot {\bf{g}}} \right],$$

is solved using a stabilized preconditioned biconjugate gradient scheme. Generalized mixed (Robin) boundary conditions are used in every dimension, i.e., d*ϕ*/d*n* + *βϕ* = 0, where *β* is adjusted on each boundary so that the derivative remains approximately constant near that boundary.

The model then evaluates the convective derivative in the continuity equation for each ion species by incorporating the ion velocities in a flux assignment scheme based on the total variation diminishing (TVD) condition^60^. We apply monotone upwind schemes for conservation laws (MUSCLs) applicable to the ion continuity problem^61^. The method combines upwind differencing schemes, flux limiting^62^, and second-order TVD schemes to minimize diffusion and dispersion in the time advance. It has been extended to 3D using a dimensional splitting technique^63^. Neumann boundary conditions are applied to the number densities on all boundaries at this stage. A second-order Runge–Kutta scheme is then used for time advance. Step sizes are 2.5 s.

The ionosphere model is initialized using plasma number densities from the Parametrized Ionospheric Model (PIM)^64^. PIM is a parametrized, physics-based ionospheric model that does not include sporadic *E* layers which, therefore, do not appear in the initial conditions of our model. Initial ion fractions are taken from the IRI-2016 model^65^. Background neutral atmospheric parameters used to calculate Pedersen, Hall, and direct conductivities and other transport coefficients are imported from the NRLMSISE-00 model^66^.

The ionosphere model is cast in magnetic dipole coordinates^47^. The grid has 192 × 161 × 145 cells in the *ϕ*, *p*, and *q* coordinates, respectively. This is an orthogonal system, simplifying model construction. In this coordinate system, most of the cross-derivative terms in the quasineutrality condition vanish identically. Further details about the coordinate system and other features of the ionosphere model were given by Hysell et al.^46,67,68^.

### Code sharing

The numerical codes used for this work are academic rather than production codes and do not currently run on publicly accessible servers. The authors will work with individuals wishing to share the codes.

### Data availability

Arecibo World-Day data are available through the Madrigal database available through https://openmadrigal.org/. Additional data products referred to in this paper are available from the authors.
